# Expanding Insurance Coverage for Pediatric Upper Extremity Recreational Prosthetic Devices

**DOI:** 10.1016/j.jhsg.2025.100790

**Published:** 2025-07-22

**Authors:** Sabrina Lazar, Gina Rose Eggert, Meera Reghunathan, Katharine M. Hinchcliff

**Affiliations:** ∗ Albany Medical College, Albany NY; † Department of Surgery, Division of Plastic Surgery, University of California San Diego, San Diego, CA

**Keywords:** Insurance, Pediatric, Hand surgery, Prosthetic

## Abstract

Pediatric limb differences often necessitate prosthesis use to assist with activities of daily living. Daily prosthetics are not designed for creative or skill- and strength-based physical activities, which has prompted the use of activity-based or “recreational” prosthetics. Pediatric activity-specific prosthetics are expensive but provide considerable physical and psychological benefits, such as increased strength, weight loss, better self-esteem, social inclusion, and higher quality of life. Currently, activity-specific prostheses are generally not covered by insurance, posing considerable financial burden in conjunction with ongoing prosthetic replacements and additional medical care. Many states are introducing and adopting policies about expanding insurance coverage for activity-specific prostheses, which are cost-saving in the long-term. To best serve our patients with limb differences, hand surgeons should advocate for expanded coverage of activity-specific prostheses for pediatric patients at local, state, and national levels.

Pediatric limb differences are associated with considerable emotional and physical stress, decreased self-esteem, and quality of life.^1^ Pediatric upper extremity (UE) loss or anomalies can be because of numerous factors, such as congenital or syndromic malformations, trauma, or malignancy. The incidence of congenital UE limb differences is estimated to be 2–3 times greater than that in the lower extremity.^2^ There is a relative paucity of recent data on pediatric UE limb differences; in the United States, approximately 1 in 2,800 children will have a congenital UE difference, and 1 in 10,000 live births will present with a transverse UE deficiency.^3^ Incidence studies on congenital limb deformities are challenging to perform because of the large population in the United States and the lack of a centralized congenital limb deformity registry. The Centers for Disease Control and Prevention itself only tracks “upper and lower limb reduction deficits” together, and approximately one in every 2,100 children are born with a limb deficit in the United States.^4^ Prostheses can be exorbitantly expensive, with body-powered prostheses averaging $4000 to $8000 and $25,000 to $50,000 for myoelectric prostheses.^5^ This article aims to describe the current barriers to accessing pediatric UE prostheses and propose action items for hand surgeons in advocating for pediatric patients with limb differences. We seek to highlight the disparity of insurance coverage of activity-specific prostheses, which are essential for facilitating play, self-esteem, and physical and social development.

### Activity-specific prostheses increase the quality of life in pediatric patients

Daily prostheses are generally designed to aid users in activities of daily living (ADLs) such as walking, standing, reaching and grasping for items, facilitating bimanual tasks, or manipulating simple tools. These, however, are often unfit for creative or high-level physical activities and can lead to mechanical failure, compensatory movements, and overuse injuries if the user repeatedly attempts these activities with the incorrect prosthesis.^6^^,^^7^ Activity-specific prostheses allow the users to pursue life interests and safely engage with their environment. These prostheses are designed for skill- and strength-based activities such as archery, peeling vegetables, fishing, and playing musical instruments. Despite their importance, recreational or activity-specific prostheses are equally expensive and excluded from most insurance plans (Fig. 1A–D).^2^^,^^8^^,^^9^ Unfortunately, if the child already has a primary prosthesis, insurers generally do not cover an additional prosthetic under the determination that it is not medically necessary.^2^ In a 2023 study of 1,566 respondents under the age of 18 years, 65% did not have a secondary prosthesis.^6^ Combined with the known high costs and scant insurance coverage of primary and recreational prostheses, these data potentially point toward access issues and user demographics. There is also a known high prosthetic abandonment rate for daily prostheses because users often learn to manage their ADLs without them. However, there are no data on the abandonment of specialized activity-related prostheses. Since activity-related prosthetics are inherently designed for more precise or complex actions, it is unknown to what extent users adapt to those movements without the prosthetic. Also, since they allow the user to participate in enjoyable recreation, the rate of abandonment may be less than in daily prosthetics, highlighting the need for more research.

**Figure 1:**
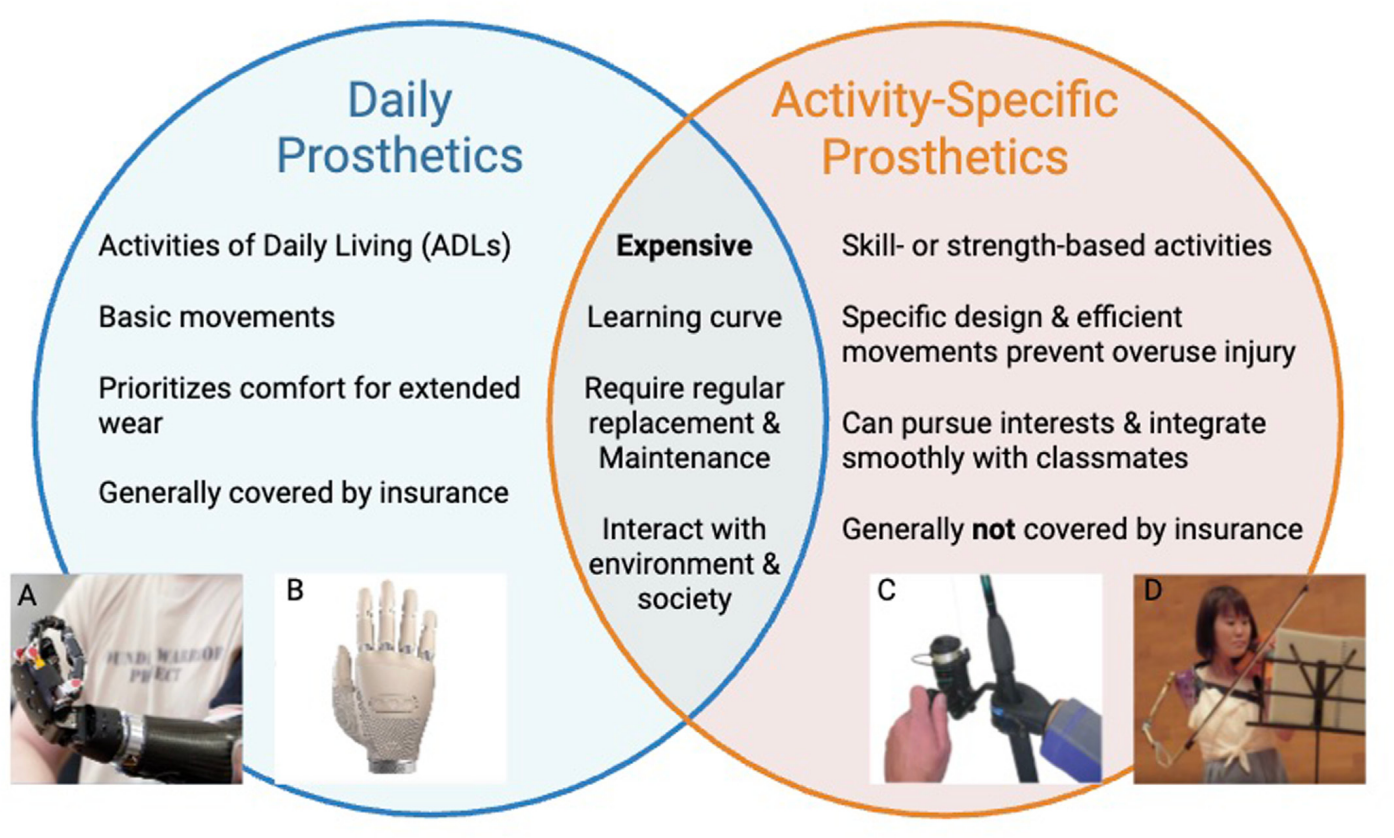
Daily versus activity-specific prosthetics. **A, B** and **C** are examples of commercially available pediatric prosthetics. **D** Depicts Manami Ito, an accomplished 11-year-old violinist using her activity-specific prosthetic. Figure created with BioRender.

Exercise and sports participation is an important part of pediatric and adolescent development and contributes to psychological and physical well-being.^10^ Children with chronic disease and physical disabilities have been found to have lower levels of physical activity and fitness and a higher prevalence of obesity compared with those without disabilities.^11^ Additionally, they are less likely to engage in sports and frequently experience low self-worth and quality of life.^11^

Adaptive pediatric athletes have demonstrated increased muscle strength, aerobic fitness, and agility associated with sports participation.^12^ In a 2021 study, the authors designed 3-dimensional printed activity-specific prostheses for golfing, cycling, and playing the cello and violin and found that they led to increased patient satisfaction and wrist strength of the affected limb after 8 weeks of use.^13^ Another small study of children using activity-specific prostheses found improved performance and skill in bimanual activities such as playing the violin or lifting barbells. This supports the fact that prosthesis users can reach proficiency by practicing their skill, like any other artist or athlete.

Promoting and expanding adaptive sporting programs for children with limb differences supports the goal of destigmatizing and normalizing the idea that children with disabilities can have fun and play sports and instruments like their peers. Most general prostheses allow basic movements for ADLs but have limited functionality outside of this. People of all ages, and especially growing children, should be able to play and interact with their environment with activity-specific prostheses and experience social activities and a higher quality of life.

### Costs and insurance coverage for UE prosthetics

Costs vary between UE prostheses, ranging from $4,000 to $75,000.^14^ Limb differences in childhood often necessitate long-term prosthetic use, which must be repaired and replaced every few years as the child grows. Despite clear medical necessity, these prostheses are not always covered by insurance. Similar inequities in prosthetic coverage exist in Canada and Germany.^15^^,^^16^ Published data on recreational prosthesis insurance coverage patterns are sparse, especially for UE and pediatric devices. The authors discussed this topic with certified orthotist-prosthetists experienced in these areas, who reported observing a discrepancy between medical payors versus private insurances; many medical coverage plans often do not reimburse for activity-specific prostheses. The billing code typically used is “L6704 Terminal Device, Sport/Recreational/Work Attachment, Any Material, Any Size” for a sports or music adapter. Each insurance plan specifies what is covered, but differences arise in the contracted rate or service reimbursement amount. For example, an insurance payor may state the L6704 code is covered, and they will authorize the claim, but the contracted rate or service reimbursement is $300, whereas the bike handlebar adapter costs the prosthetist $500 to purchase from the manufacturer. The interviewees further comment that Shriners and other community payors generally have low service reimbursement rates, or patients’ families must pay of pocket, exacerbating the cost-prohibitive nature of these devices.

Additionally, many private insurers have implemented yearly and lifetime spending and outpatient office visit limits. In 2012, yearly caps for prosthesis-related charges were maximized at $3000/year, and some policies only covered a single device over the person’s lifetime.^14^ Children and adolescents experience rapid limb and body development, which can require frequent visits to optimize prosthetic fit as they grow. Experiencing high costs for prostheses is the most common factor impeding sports participation and regular prosthetic replacement and maintenance.^12^

### Financial impact and current advocacy efforts

Over 50 nonprofits currently donate orthotics and prosthetics, but they cannot cover the estimated 2 million people living with limb loss.^17^ To address this disparity, the “So Every BODY Can Move” initiative is involved in implementing policies and legislation to increase insurance coverage of recreational prosthetics for all ages. Mainly led by orthotists and prosthetists, it has successfully enacted legislation for coverage of medical and recreational prosthetics in Maine, Arkansas, Colorado, and New Mexico. As of 2023, bills have been introduced in Illinois, Indiana, Minnesota, New Jersey, and New Hampshire, and they continue broadening their efforts.

Critics of expanding insurance coverage warn of a potential subsequent and considerable increases in taxpayer burden; anticipating this, Kehoe et al^9^ provided a thorough analysis of the fiscal and social impact of these new activity-specific prosthesis policies. They calculated the per person per month costs after implementing the expanded insurance coverage would increase by only $0.012 in Maine, and the estimated spending increase to Colorado, Connecticut, and Illinois combined would be less than 0.003% of the annual health care per capita cost in the United States, which is $10,000.^9^ Their findings suggest that there will be long-term cost-saving associated with each bill to cover activity-specific prostheses, since lower-extremity problems resulting from inappropriate prosthetic use can range from $80,000 to $150,000 over an individual’s lifetime.^9^ In 2015 alone, the annual cost of prostheses and medical care for children with lower limb loss equaled $206.8 million.^1^ Preventing compensatory overuse injuries and other issues from attempting high-level activities with the wrong prosthesis will save costs for both individuals and taxpayers.^9^ In addition, activity-specific prostheses will allow children to experience recreational activities, maintain healthy lives, and benefit from increased psychosocial and physical wellness. In 2013, the US Department of Education Office for Civil Rights mandated school districts that receive federal funding are legally obligated to provide sports programs for students with disabilities.^2^ These adaptive recreational opportunities represent a considerable missed opportunity for wellness among the pediatric population that does not have access to appropriate, well-fitted prosthetics.

Further work is needed to resolve the disparity of children with limb differences not being able to experience sports enrichment and the psychosocial benefits of participation in activities. One specific area of interest is in the definitions of “prosthetic device” and “medically necessary.” The definition of “prosthetic device” adopted by many insurers does not include a device made specifically for athletic or recreational use.^9^ Arizona, Colorado, Utah, and California do not specify whether a prosthetic must be deemed medically necessary for insurance coverage.^9^ The current definition of “medically necessary” used by insurers does not include activity-specific prostheses, despite overwhelming data on their benefits to quality of life, specifically in physical and behavioral health. Therefore, some advocates are working to have these definitions expanded or redefined appropriately. Because of their clear positive impact on a child’s mental and physical health in the form of improved psychosocial development, self-esteem, physical exercise, and motor skills, we posit that activity-specific prostheses reasonably fall under the definition of “medically necessary” to pediatric patients living with limb differences.

Policies do not necessarily need to cover multiple activity-specific prostheses that a child wishes to have across a spectrum of activities; this may be cost-prohibitive and inhibitory to advocacy efforts. However, even covering one activity-specific prosthesis in addition to a general prosthesis will allow a child to participate in creative and physical activities instead of being socially and physically isolated because of disability.

### How surgeons can lead advocacy efforts

Collaboration and provider education is key to success in caring for pediatric patients with limb differences. Surgeon advocacy can include working with a multidisciplinary team of physiatrists, prosthetists, and others supporting each child’s journey with limb deficiency or deformity (Fig. 2). A 2022 survey of the American Association of Hand Surgery members found that hand surgeons are overall unfamiliar with modern partial hand prosthetic devices, and 76% neither consulted a prosthetist before revisional UE surgeries nor collaborated with a multidisciplinary hand team.^18^ Collaborating with prosthetists and other health care providers promotes coherent, consistent communication between all team members and allows the patients’ goals and concerns to be met efficiently. We encourage hand surgeons to leverage their medical knowledge and firsthand experience with social determinants of health to advocate for the physical and psychosocial well-being of their pediatric patients through increased access to activity-specific prostheses. Already several states have introduced and passed pieces of legislation calling for activity-specific prostheses to be included under insurance coverage.

**Figure 2:**
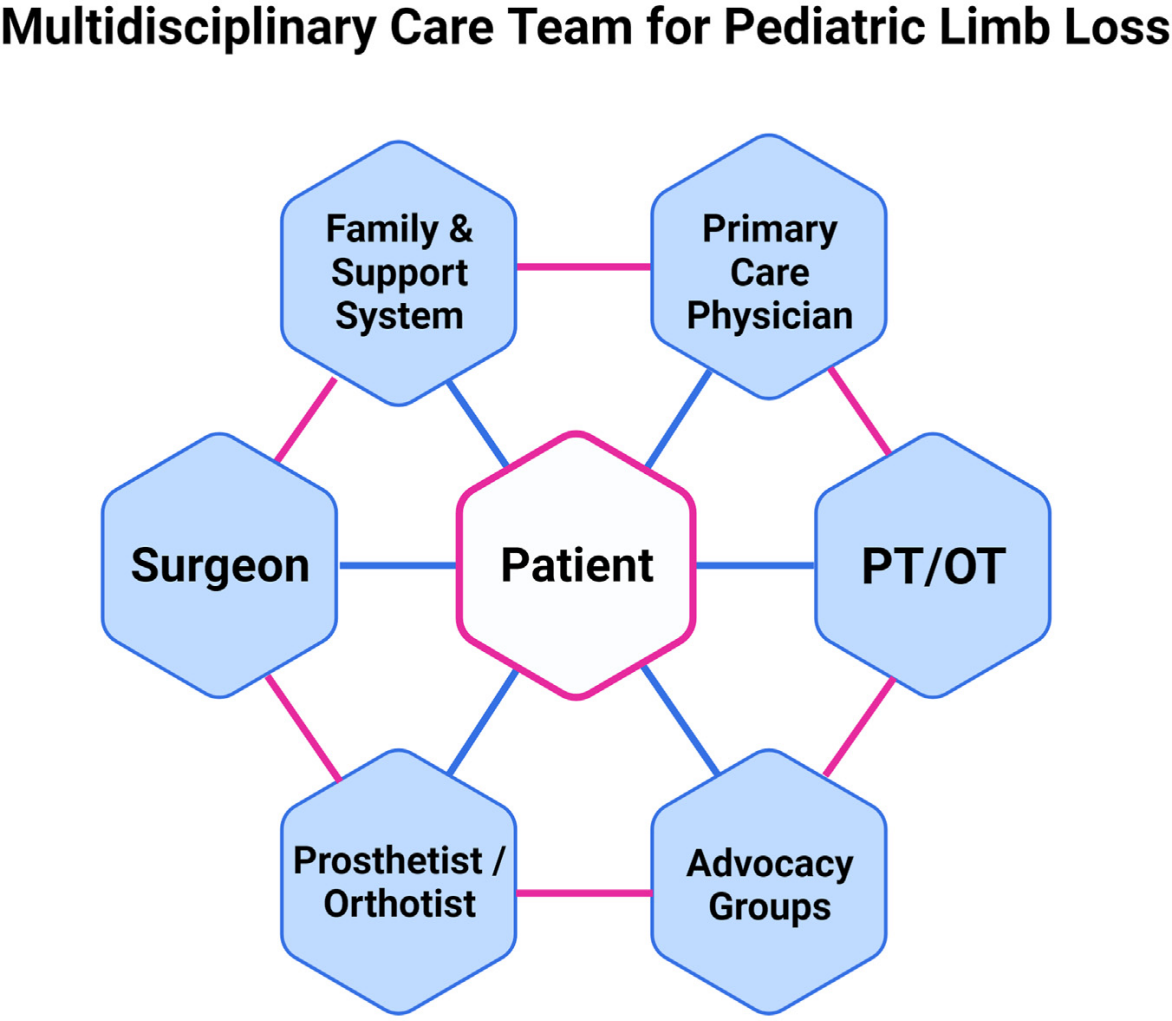
Diagram depicting the interconnected network between each of the many physicians, providers, and other key individuals all working to support and care for each pediatric limb loss patient. PT/OT, physical therapy and occupational therapy. Figure created with BioRender.

Other ways to partake in advocacy include joining state, regional, or national specialty societies and speaking up at meetings, calling the local senator’s office to notify them of issues in the community, becoming a trusted media liaison on health issues, and leveraging the power of writing to spread awareness.^19^ By spending a few hours each month, collaborating with strategic partners, and developing a realistic action plan, physicians across specialties can create institutional and policy-level change. Physicians can also play meaningful roles in nonprofit leadership, speak at community events, and attend lobby days in the local government alongside coalitions or specialty-specific societies to use collective bargaining power and political capital. Surgeons can join the Political Action Committee (PAC) for regional and national organizations to impact policy at the state and national levels. The Orthopedic PAC by the American Academy of Orthopedic Surgeons and the PlastyPAC by the American Society of Plastic Surgeons are examples of these advocacy groups. Seemingly small or individual-led efforts that begin now may lead to a “snowball” effect in the future as strides are made, and others join the cause.

## Future Direction

Notable disparities in access and coverage remain among pediatric patients living with extremity differences. Work remains to expand this movement and potentially advocate for insurance companies to amend the term “prosthetic” device to include activity-specific prostheses or redefine “medically necessary” to include prosthetics designed specifically for athletic and recreational purposes, given their considerable positive impact on health, fine and gross motor skills, and well-being. Physicians can raise these concerns to local policymakers or join one of the many nonprofits making community-level differences. Additionally, we call on orthopedic and plastic surgeons to work closely with physiatrists and prosthetists to ensure comprehensive, cohesive care for our patients with limb differences.

## Conflicts of Interest

No benefits in any form have been received or will be received related directly to this article.
